# Advanced Oxidative Protein Products Had a Diagnostic Accuracy for Identifying Chronic Kidney Disease in Adult Population

**DOI:** 10.3390/metabo14010037

**Published:** 2024-01-07

**Authors:** Diana Carolina Villalpando-Sánchez, César Arturo Barajas-Medina, Cleto Alvarez-Aguilar, Geovani López-Ortiz, Luisa F. Romero-Henríquez, Anel Gómez-García

**Affiliations:** 1 Posgrado en Inmunología, Escuela Nacional de Ciencias Biológicas, Instituto Politécnico Nacional, Mexico City 11350, Mexico; dvillalpandos1901@alumno.ipn.mx; 2 División de Investigación Clínica, Centro de Investigación Biomédica de Michoacán, Instituto Mexicano del Seguro Social, Morelia 58341, Mexico; 3 Unidad de Medicina Familiar No. 80, Instituto Mexicano del Seguro Social, Morelia 58000, Mexico; cesar.medina@imss.gob.mx; 4 Facultad de Ciencias Médicas y Biológicas “Dr. Ignacio Chávez”, Universidad Michoacana de San Nicolás de Hidalgo, Morelia 58000, Mexico; cleto.alvarez@umich.mx; 5 Subdivisión de Medicina Familiar, Facultad de Medicina, Universidad Nacional Autónoma de Mexico, Ciudad de Mexico 04510, Mexico; geovani.lorz@fmposgrado.unam.mx; 6 Posgrado en Pedagogía, Facultad de Filosofía y Letras, Universidad Nacional Autónoma de Mexico, Ciudad de Mexico 04510, Mexico; fer_55rh@comunidad.unam.mx

**Keywords:** advanced protein oxidation products, glomerular filtration rate, diabetes mellitus, type 2, chronic kidney disease, diagnostic accuracy

## Abstract

Chronic Kidney Disease (CKD) is a serious public health problem. Hyperglycemia stimulates the production of reactive oxygen species that cause oxidative damage to proteins. AOPPs constitute a group of oxidized dityrosine-containing proteins that are generated during periods of oxidative stress. They have proved to be a valuable early marker of oxidative tissue damage and active mediators of inflammation associated with the uremic state. To analyze if advanced oxidative protein products (AOPPs) have diagnostic accuracy for identifying chronic kidney disease (CKD) in the adult population. We conducted a diagnostic test validation study in 302 adults ≥20 years old, of both sexes, with and without T2D. After obtaining informed consent, a comprehensive clinical history, anthropometric measurements (weight, BMI) and blood pressure were recorded. Glucose, cholesterol, triglyceride, HDL-c, LDL-c and AOPPs were determinates. Glomerular filtration rate (GFR) was calculated using Cockcroft–Gault (C–G) corrected by body surface area (BSA, mL/min/1.73 m^2^), CKD-EPI and MDRD equations to identify five stages of CKD. This study follows the Standards for Reporting Diagnostic Accuracy Studies (STARD). The median value of AOPPs was 198.32 µmol/L (minimum-maximum value: 113.48–522.42 µmol/L). The group with patients diagnosed with T2D exhibited higher concentrations (median: 487.39 µmol/L) compared to the non-diabetic group (median: 158.50 µmol/L, *p* = 0.0001). The selected cut-off point was ≥200 µmol/L using the closest to the median value of AOPPs with sensitivity and specificity as follows: C–G: sensitivity 96.58%; specificity 80%; likelihood ratio: 4.83; CKD-EPI: sensitivity 95.76%; specificity 79.89%; likelihood ratio: 4.76; MDRD: sensitivity 86.55%; specificity: 73.22%; likelihood ratio: 3.23. A difference was observed between AOPPs and chronic kidney disease stage. This study provides evidence that AOPPs ≥ 200 µmol/L have diagnostic accuracy in identifying stage 4–5 CKD by C–G, MDRD and CKD-EPI equations in adults with and without T2D.

## 1. Introduction

Chronic kidney disease (CKD) is a serious public health problem in developing countries and presents a crucial challenge in global health policies due to its detrimental effects on quality of life. It reduces overall well-being, diminishes survival rates, independence and productivity and it adds an economic burden to institutions in Mexico. Risk factors associated with lifestyle, such as stress, a sedentary lifestyle and malnutrition, play a role in the development of obesity and metabolic disorders (such as hyperglycemia, dyslipidemia and hyperuricemia), which impair kidney function and thus, increase the risk of CKD development [1,2].

It is known that an obese phenotype unassociated with metabolic abnormalities per se predicts a higher risk of incident CKD [3]. Despite the widespread adoption of glucose-lowering therapies and renoprotective therapies, patients with diabetes remain at high risk of development of end-stage renal disease [4]. Identification of pathological processes that lead to an early loss of glomerular filtration rate (GFR) represents an opportunity for the implementation of timely interventions in the first level of attention.

Microalbuminuria and glomerular filtration rate (GFR) are classically used to evaluate an individual’s kidney function and stage the progression of chronic kidney disease. GFR measurement is crucial to account for pharmacological prescriptions [5]. Numerous equations have been developed to estimate GFR in adults who use creatinine and include age, sex, race and body size. The formulas used to estimate GFR in primary care medical units have been the Cockcroft–Gault (C–G) corrected by body surface area (BSA, mL/min/1.73 m^2^) equation and, currently, Modification of Diet in Renal Disease (MDRD) and Chronic Kidney Disease Epidemiology Collaboration (CKD-EPI) [6].

Additionally, chronic hyperglycemia activates metabolic routes that involve diacylglycerol, protein kinase C and NADPH-oxidase stimulating the excessive production of reactive oxygen species (ROS) such as superoxide radicals (O_2_^•-^), hydrogen peroxide (H_2_O_2_) and hydroxyl radicals (OH^•^). These highly reactive molecules can cause damage to proteins, making them oxidative targets. Exposure to these molecules leads cells to a state of oxidative stress (OS) and tissue damage that is a condition characterized by an imbalance between the production of ROS. This imbalance can overwhelm the body’s ability to neutralize these harmful molecules, resulting in oxidative damage to cellular components such as proteins, lipids and DNA [7,8]. One of the biomarkers used to quantify OS is advanced protein oxidation products (AOPPs), which serve as reliable indicators of tissue damage resulting from OS [9]. AOPPs serve as crucial oxidant biomarkers, offering valuable insights into oxidative damage inflicted upon proteins within biological systems. AOPPs constitute a group of oxidized dityrosine-containing proteins that are generated during periods of oxidative stress. They have proved to be a valuable early marker of oxidative tissue damage and active mediators of inflammation associated with the uremic state [10]. Monitoring AOPP levels not only provides insights into the extent of oxidative damage but also serves as a potential avenue for understanding and managing inflammation in the context of uremia [11]. Furthermore, it is estimated that increased concentrations of these products could play a role in the pathogenesis and progression of complications related to diabetes and diabetic nephropathy [12,13,14]. There was no established cut-off point in the literature for studies in patients with chronic kidney disease (CKD).

The objective of this study was to analyze whether AOPPs have diagnostic accuracy in identifying CKD in adults with and without T2D.

## 2. Materials and Methods

We conducted a prospective diagnostic accuracy study from January to December 2021 on adults of the Family Medicine Unit (FMU) N° 80 of the Mexican Institute of Social Security (IMSS) in Morelia, Michoacán, Mexico.

### 2.1. Sample Size

A sample size for testing the sensitivity of the single diagnostic test was realized [15]. We used a 95% confidence level and 80% of power to detect a difference of 7% and a presumption value of sensitivity of 73%, obtaining a sample size of at least 296 participants.

### 2.2. Participants

The participation of subjects was voluntary and was based on an invitation by the primary care doctor under the criteria that they were adults over 20 years, of either sex, with or without T2D and that they wished to participate in the study. A total of 392 adults were collected from the FMU outpatient clinic and selected by a family doctor in accordance with the inclusion criteria. All participants were provided with a comprehensive explanation to elucidate the study’s purpose and the methodologies employed, and they provided their informed consent by signing document. This study adheres to the Standards for Reporting Diagnostic Accuracy Studies (STARD) [16].

The inclusion criteria for all patients were that they were adults over 20 years old and of either sex. For patients diagnosed with T2D between 2 and 30 years (with a median duration of 15 years), 73% were treated with oral hypoglycemic agents, 9% with insulin alone and 18% with a combination of insulin and oral hypoglycemic agents.

The exclusion criteria were subjects with a history of viral infection, drug usage, inflammatory processes or those undergoing pharmacological treatment during the previous month because this can interfere with the formation of AOPPs. Ninety patients were subsequently excluded due to prior infections or incomplete laboratory studies. Ultimately, 302 subjects were included in the study.

For each participant, a comprehensive clinical history was gathered, along with anthropometric measurements and blood pressure assessments. Weight was measured to the nearest 0.1 kg and height was measured to the nearest 0.1 cm. Body Mass Index (BMI) was calculated according to the Quetelet index [weight (kg)/height (m^2^)]. Blood pressure was recorded using a sphygmomanometer (Welch Allyn^®^ Mod WA7670-10, NY, USA) after 20 min of rest, with the participant in a supine position. A suitable cuff was placed on the dominant arm following the International Criteria of JNC VIII [17].

### 2.3. Biochemical Parameters

Blood samples were collected by a phlebotomist in the clinical laboratory of the FMU N° 80, IMSS following WHO guidelines on drawing blood: best practices in phlebotomy [18]. After a 12 h fasting period from a vein in the antecubital fossa without venous occlusion, 6 mL were obtained in one tube with gel separator 13 × 100 mm (BD Vacutainer^®^ Blood Collection Tube; Becton Dickinson, and Company, Manufactured by Becton Dickinson and Company, Sumter, SC, USA) and 3 mL in tube with K_2_EDTA of 13 × 75 mm (BD Vacutainer^®^ Collection Tube; Becton Dickinson, and Company).

After centrifugation of the tube with gel separator (1500× *g* for 5 min) by sera obtention, glucose, total cholesterol (TC), triglyceride (TG) and high-density lipoprotein cholesterol (HDL-c), creatinine concentrations were measured immediately using an automated analyzer [Vitros^®^ 350 Chemistry System Ortho Clinical Diagnostics (Raritan, NJ, USA)]. These determinations were performed at the clinical laboratory of the FMU N° 80, IMSS. The intra-analysis coefficient of variation for these tests was 1%

The tube with K_2_EDTA was centrifugated (600× *g* × 10 mm) and the plasma was stored in 500 μL aliquots in transparent sterile polypropylene microtubes with snap-on lids (Axygen^®^ Scientific, Corning Incorporated, Corning, NY, USA) at −70 °C until analysis to ensure minimal inter-assay variability before subsequent analysis for AOPPs’ quantification.

Low density proteins cholesterol (LDL-c) were calculated using the Friedewald equation [LDL-c (mg/dL) = Total Cholesterol (mg/dL) − HDL-c (mg/dL) − (Triglycerides (mg/dL)/5)]. Glomerular Filtration Rate, (GFR) was calculated by Cockcroft–Gault (C–G) corrected by body surface area (BSA, mL/min/1.73 m^2^) equation, MDRD and CKD-EPI all as reference standard [19].

In all participants, both adults with and without T2D, the classification of CKD involves distinguishing between five stages based on the GFR: (stage 1) characterized by a normal or increased GFR (GFR ≥ 90 mL/min/1.73 m^2^); (stage 2) marked by the coexistence of kidney damage alongside a slightly decreased GFR (GFR: 60–89 mL/min/1.73 m^2^); (stage 3) involving a moderate decrease in GFR (GFR 30–59 mL/min/1.73 m^2^); (stage 4) indicating a severe reduction in glomerular filtration rate (GFR 15–29 mL/min/1.73 m^2^); and (stage 5) requiring the initiation of supportive treatments such a dialysis or transplant due to a GFR less than 15 mL/min/1.73 m^2^ [20].

### 2.4. AOPPs Determination

Plasma concentrations of AOPPs were determined according to the spectrophotometric assay described by Witko-Sarsat et al. [9]. First, a Chloramine T solution was prepared at a concentration of 100 μM for the calibration of the standard curve. To do this, 0.0031 g of Chloramine T hydrate [Lot #125K0731 from Sigma-Aldrich Chemical Company, St. Louis, MO, USA] was weighed and dissolved in 100 mL of distilled water. Second, a KI solution was prepared with a concentration of 1.16 M with 1.925 g of potassium iodide [Lot #0001437438 from Sigma-Aldrich], and dissolved in 10 mL of distilled water. Third, the PBS solution for the blank was prepared by weighing 10.2 g of PBS Buffer Powder [Lot #0608815 from Sigma-Aldrich Chemical Company, St. Louis, MO, USA], which was then dissolved in 1 L of distilled water.

### 2.5. Calibration Curve

To perform the calibration curve, we started with the Chloramine T solution at 100 μM (Tube 1). Tube 2 (Chloramine T 50 μM) was prepared with 400 μM of Chloramine T, and 100 μM is supplemented with 400 μM of distilled water. Tube 3 (Chloramine T 25 μM) was prepared by taking 400 μL from tube 2 and adding 400 μL of distilled water. Tube 4 (Chloramine T 12.5 μM) was prepared by taking 400 μL from tube 3 and adding 400 μL of distilled water. Tube 5 (Chloramine T 6.25 μM) was prepared by taking 400 μL from tube 4 and adding 400 μL of distilled water. Tube 6 (Chloramine T 3.125 μM) was prepared by taking 400 μL from tube 5 and adding 400 μL of distilled water. Tube 7 (Chloramine T 1.562 μM) was prepared by taking 400 μL from tube 6 and adding 400 μL of distilled water. Tube 8 (Chloramine T 0 μM) was prepared by taking 400 μL from tube 7 and adding 400 μL of distilled water, and 400 μL was mixed and withdrawn from the tube. To each of the previous solutions, 40 μL of Acetic Acid [Lot #01315KE Sigma-Aldrich Chemical Company, St. Louis, MO, USA] was added at 99%, and 20 μL of 1.16 M KI solution was added. This was stirred for a few seconds and immediately read on the spectrophotometer Synergy HT, Biotek^®^ (MA, USA) at a wavelength of 340 nm. The chloramine-T absorbance at 340 nm exhibited a linear relationship within the range of 0–100 µmol/L.

### 2.6. Sample Analysis

Once the corresponding curve had been prepared, the collected plasma samples were analyzed by diluting 200 μL of plasma at a 1:5 ratio in PBS solution. From this dilution, 400 μL was taken, to which 40 μL of acetic acid and 20 μL of 1.16 M KI were added, respectively. The sample’s absorbance was read immediately on the spectrophotometer Synergy HT, Biotek^®^ at a wavelength of 340 nm. The resultant AOPPs concentration was multiplied by the dilution factor (Concentration × 5) and expressed in micromoles per liter (mol/L) of chloramine T equivalents. The intra- and inter-assay coefficients of variation (CV) obtained via this methodology in our laboratory were both under 5%.

### 2.7. Ethical Considerations

This study was authorized with number R-2020-1602-017 by the Research Ethics Committee No. 16028 and the Local Health Research Committee No. 1602 of HGR No. 1 of Mexican Institute of Security Social (IMSS) and was governed by the ethical considerations of the Declaration of Helsinki, the Universal Declaration of Bioethics and Human Rights and the Regulation of the General Law of Health in Research for Health.

### 2.8. Statistical Analysis

The Kolmogorov–Smirnov test was used to assess the normality of distribution of the investigated parameters. All variables were distributed abnormally and the data were expressed as median and minimum–maximum values. For the comparison of biochemical variables between the groups of patients divided by gender, with and without diabetes, the Mann–Whitney U test was employed. Gender distribution within the groups of patients with and without diabetes was assessed using Pearson’s chi-square. Spearman’s Rho Correlation Coefficients were used to analyze the bivariate relationships. The AOPPs’ concentration at different stages of CKD was realized by the Kruskall–Wallis test.

Receiving Operating Curves (ROC) was performed to estimate the sensibility, specificity and odds ratio of the cut-off point of AOPPs by C–G, MDRD and CKD-EPI equations. All tests were two sided. For the reliability of AOPPs, we calculated the positive predictive value (probability of being in stage 4–5 of CKD if a result of ≥200 µmol/L or higher in AOPPs was obtained) and the negative predictive value (probability of a subject being in stages 1–3 of CKD with a result <200 µmol/L in AOPPs). The data was analyzed in the statistical package SPSS version 23. Differences were regarded as significant if *p*-value < 0.05.

## 3. Results

**Figure 1:**
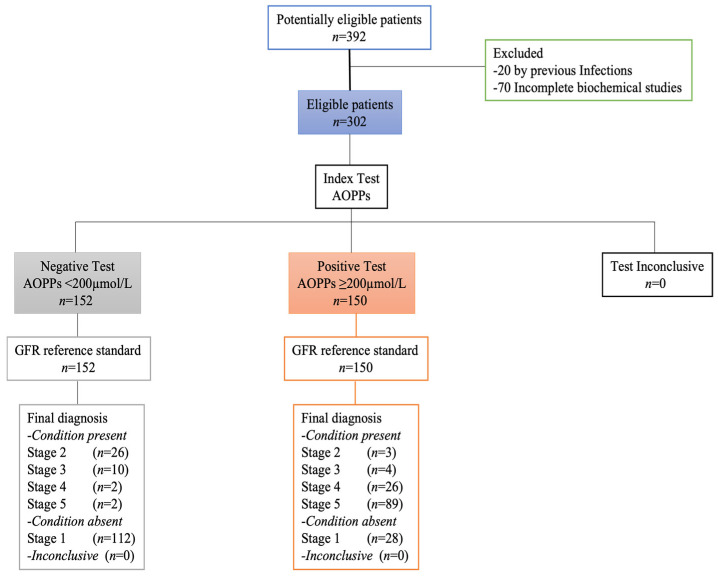
STARD diagram of participants.

Between 15 January and 15 December 2021, 392 adults with and without T2D were selected, of whom 90 were excluded due to previous infections (*n* = 20) and incomplete clinical studies (*n* = 70). A total of 152 adults had a negative test for AOPPs with a cut-off point <200 µmol/L, and 150 adults had values above 200 µmol/L, which was considered positive for AOPPs. All selected adults underwent the tests related to this study. The STARD diagram of adults throughout the study was reported in Figure 1.

A total of 302 adults were included in this study. Baseline demographic and clinic characteristics are presented in Table 1. The distribution of the disease was 213 individuals with T2D and 89 without T2D. The median age of individuals with T2D was 60 years (range: 28–75), with 39.9% female and 60.1% male participants. Of these, 23% had a normal weight, 46.9% were overweight and 30% were classified as obese. The median age of individuals without T2D was 60 years (range: 28–75), with 87.6% female and 12.4% male participants. Of these, 19.1% had a normal weight, 49.4% were overweight and 31.5% were classified as obese.

The median value of AOPPs was 198.32 µmol/L (minimum–maximum value: 113.48–522.42 µmol/L). We compared AOPPs between adults with and without T2D. In this regard, the group with T2D had statistically significantly higher concentrations (487.39 µmol/L; minimum–maximum value: 115.57–542.68 µmol/L) than the group without T2D (158.50 µmol/L, 110.40–212.10 µmol/L *p* = 0.0001). Additionally, we compared AOPPs by sex between adults with and without T2D. The group with T2D, woman had higher concentrations (493.55 µmol/L; minimum–maximum value: 137.40–522.42 µmol/L) than the men (340.98 µmol/L; minimum–maximum value: 113.63–518.68 µmol/L) with non-statistically significant gender-related (*p* = 0.860). The group without T2D, woman had higher concentrations (162.26 µmol/L; minimum–maximum value: 113.44–212.10 µmol/L) than the men (110.67 µmol/L; minimum–maximum value: 92.25–170.99 µmol/L) with non-statistically significant gender-related (*p* = 0.113).

Correlations between AOPPs and other measured variables are shown in Table 2. AOPPs were positively associated with creatinine value (Spearman Rho: 0.754; *p* = 0.0001) and negatively associated with GFR (Spearman Rho: −0.826; *p* = 0.0001).

We proceeded to classify them by renal stage according to the GFR. The value of AOPPs by C–G corrected by body surface area (BSA, mL/min/1.73 m^2^), MDRD and CKD-EPI equations throughout the different stages is reported in Table 3. The AOPPs’ concentration by renal stage and AOPSs in stages 4 and 5 were significantly higher compared to stages 1 to 3 (*p* = 0.0001) with three equations. AOPPs were no different between stages 1 versus 2 and 3.

Until now, there has been no cut-off point for AOPPs reported in the literature. Therefore, a Receiving Operating Curve (ROC) was performed to establish the cut-off points and the predictive probability of stage 4–5 CKD based on GFR using the C–G corrected by body surface area (BSA, mL/min/1.73 m^2^), MDRD and CKD-EPI equations.

**Figure 2:**
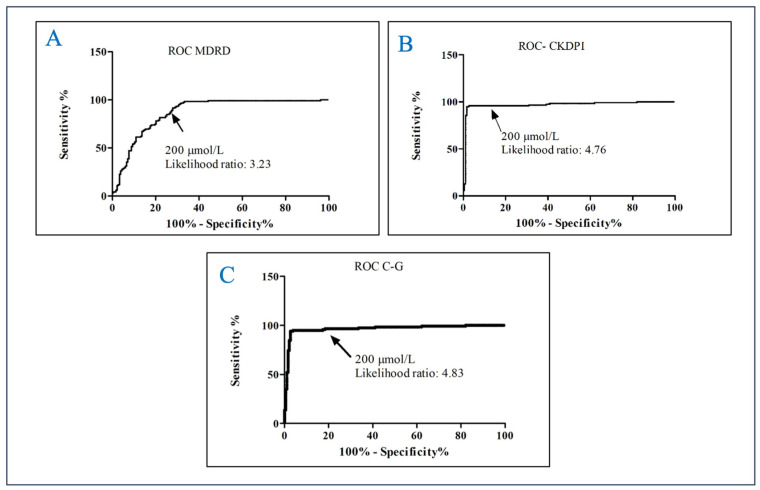
Receive Operating Curve of AOPPs for predicting kidney damage in adults. (**A**) ROC MDRD; (**B**) ROC CKDPI; (**C**) ROC Cockcroft–Gault.

The selected cut-off point was ≥200 µmol/L, using the closest to the median value of AOPPs. Using the MDRD equation yielded an AUC of 0.8710 95%CI 0.8308–0.9112, sensitivity of 86.55% and specificity of 73.22% with a likelihood ratio of 3.23, a positive predictive value of 95.79% and a negative predictive value of 80.32%. With the CKD-EPI equation, AUC: 0.9684 95% CI 0.9446–0.9921, the sensitivity was 95.76%, the specificity was 79.89%, the likelihood ratio was 4.76, the positive predictive value was 95.76% and the negative predictive value was 79.89%. With the C–G corrected by body surface area (BSA, mL/min/1,73 m^2^), equation, AUC: 0.9666 95%CI 0.9436–0.9895, the sensitivity was 96.58%, the specificity was 80%, the likelihood ratio was 4.83, the positive predictive value was 96.36% and the negative predictive value was 80.87% (Figure 2).

## 4. Discussion

The present study suggests that advanced oxidation protein products’ determination has diagnostic accuracy, exhibiting high sensitivity and specificity for detecting chronic kidney disease among patients with T2D. Adults with AOPPs ≥200 µmol/L had a 4.83 higher probability of stage 4–5 CKD as indicated by GFR calculated using the C–G equation. Similarly, using the CKD-EPI equation, the likelihood ratio was 4.76, suggesting a comparable diagnostic capacity.

Numerous factors are related with a pathological process that results in the loss of GFR in diabetes. Identifying the earliest stages of declining GFR is a primary objective in health care. This identification enables us to formulate strategies for implementing interventions when they are most likely to be effective. Furthermore, is well known that subsequent progression of microalbuminuria to macroalbuminuria is a dynamic process with prognostic importance associated with GFR decline [21]. The Clinical Laboratory of Family Medicine Units of IMSS does not currently offer a microalbuminuria test. Therefore, to assess the decline GFR, we rely on the evaluation of serum creatinine and Cockroft–Gault equation. However, recognizing this limitation, we are actively exploring alternative testing options for prognostic biomarkers that are relevant for patients with T2D. AOPPs offer several advantages due to their relatively early formation, greater stability and reliability and their longer life span [8]. Moreover, in Mexico, the cost of AOPPs per test in our laboratory is approximately USD 1 in comparison with microalbuminuria in a 24-hour urine sample, which ranges between USD 15 and 35.

Previous research has demonstrated a correlation between plasma levels of AOPPs and plasma creatinine concentration, which increased progressively with the degree of albuminuria [22]. The authors of this previous study suggested that the potential utility of AOPP measurement as a diagnostic tool for identifying diabetic nephropathy and assessing its progression. Their suggestion implies that AOPP levels may serve as indicative markers, offering valuable insights into the extent of renal complications associated with diabetes. Further research and clinical investigations are warranted to validate and explore the full scope of AOPPs as a diagnostic and prognostic tool for diabetic nephropathy.

The pathogenic role of AOPPs in kidney damage is sustained by numerous experimental observations that support our results, demonstrating consistency in elevated AOPP levels in patients with hyperglycemia and/or dyslipidemia. This outcome is an expected situation since detrimental cellular events triggered by AOPPs contributed to disruptions in renal function. Elevated glucose concentrations and the subsequent formation of advanced glycation ends products (AGEs), as well as ROS, result in the glycosylation of glomerular and tubular proteins. This process activates pathways such as Janus kinase/signal transducers/activators of transcription (JAK-STAT), nuclear factor-kB (NFkB) and p38 mitogen-activated protein kinase pathways [23,24] that decreased creatinine clearance and led to exacerbation of glomerulosclerosis and interstitial fibrosis [25,26].

In this study, AOPPs were found to be significantly higher in T2D patients compared to the No-T2D Group. Different AOPP concentrations have been reported by other authors in young, apparently healthy people at risk of cardiovascular factors [10], as well as in patients with pathologies such as endothelial dysfunction and vascular inflammation [27,28,29], diabetes [30,31], hypertension [12] and nephropathy [32,33]. These studies have presented different concentrations of AOPPs without establishing a specific cut-off point. We believe that the variations in concentrations are attributed to the omission of the dilution factor application. Thus, we emphasize the importance of incorporating the conversion factor of the dilution applied to a patient sample, aligning with the initial protocol established by Witko et al. [9], and promote access to this study in clinical laboratories.

Given the absence of an international consensus on the diagnostic utility of AOPPs, to our knowledge this study represents the first instance where a defined cut-off point has been reported. Utilizing the Cockroft–Gault equation, a cut-off point of AOPPs ≥200 µmol/L was determined, revealing a 4.83-fold increased risk of kidney disease. This value signifies a diagnostic accuracy in identifying CKD that is similar to the CKD-EPI equation. This test offers an auxiliary tool for primary care physicians to effectively identify patients with suspected kidney disease. In accordance with Heidari et al., the AOPPs test establishes its position in relation to the Cockroft–Gault, CKD-EPI and MDRD equations, which serve as the gold standard utilized in Family Medicine Units in Mexico. Due to their low cost, impressive sensitivity (96%) and specificity (80%), AOPPs are recommended as a viable option.

This study presents certain limitations. First, not all potentially eligible patients (n = 390) were assessed through the reference standard methods due to non-participation in sample collection, particularly among T2D patients, despite the importance of their condition. We tried to control all possible bias. As for selection bias, the sample included in the study is representative of the FMU N° 80 populations; a weakness is that it was not possible to match by age since it is very difficult to find a young person with T2D and an important bias in this study arises from a significant difference in gender distribution among the participants. As for verification bias, the reference test was applied to all study subjects, diagnostic confirmation of all study subjects was obtained and all diagnostic tests were applied and interpreted blindly and independently to avoid the bias of Diagnostic Suspicion.

## 5. Conclusions

This study provides evidence of AOPPs ≥200 µmol/L had diagnostic accuracy to identify stage 4–5 CKD with C–G, MDRD and CKD-EPI equations in the adult population with good sensitivities, specificities, a positive predictive value and negative predictive value in adults with and without T2D. Additional multicenter studies are needed to establish the effectiveness of the AOPP cut-off point in patients with T2D and other comorbidities that lead to decreased GFR.

## Figures and Tables

**Table 1:** Baseline demographic and clinical characteristics of adults.

VARIABLES	With DM2 *n* = 213	Without DM2 *n* = 89	*p*
Age (years)	60 (28–75)	40 (21–56)	0.0001
Gender (woman/men)	85/128	78/11	0.0001
SBP (mmHg)	120 (110–130)	110 (100–120)	0.001
DBP (mmHg)	80 (60–90)	80 (50–90)	0.001
Weight (kg)	69 (60–114)	68.9 (61.5–78)	0.887
BMI (kg/m^2^)	27.1 (25–34.4)	28.4 (18.03–31.7)	0.140
Glucose (mg/dL)	133 (101.2–168.5)	88.9 (32–96.3)	0.0001
TC (mg/dL)	168 (131–338)	192 (55–220.92)	0.0001
TG (mg/dL)	157 (127–298.36)	167 (60–223.4)	0.240
LDL-c (mg/dL)	98 (90–122)	132.7 (104–238)	0.0001
HDL-c (mg/dL)	48 (25–80)	23 (12–55)	0.0001
Creatinine (mg/dL)	3.35 (0.8–10.01)	0.7 (0.5–0.9)	0.0001
AOPPs (µmol/L)	487.3 (115.57–522.42)	158.5 (113.4–212.10)	0.0001

SBP: systolic blood pressure; DBP: diastolic blood pressure; BMI: body mass index; TC: Total Cholesterol; TG: triglycerides; LDL-c: low density lipoproteins cholesterol; HDL-c: high density lipoproteins cholesterol. Median (Minimum and maximum value). Mann–Whitney U test. *p* < 0.05. Gender was analyzed with Pearson’s chi-square.

**Table 2:** Correlations between AOPPs and biochemical variables in adults.

	SBP	DBP	Glucose	TC	TG	Cr	GFR
AOPPs	0.401 **	0.159 **	0.168 **	−0.390 **	0.009	0.754 **	−0.826 **
SBP		0.609 **	0.163 **	−0.138 **	−0.038	0.331 **	−0.383 **
DBP			0.117 *	−0.112	0.026	0.082	−0.102
Glucose				0.018	0.172 **	0.009	−0.098
TC					0.401 **	−0.295 **	0.335 **
TG						0.053	0.060
Cr							−0.734 **

AOPPs: Advanced Oxidative Protein Products; SBP: Systolic Blood Pressure; DBP: Diastolic Blood Pressure; TC: Total cholesterol; TG: Triglycerides; Cr: Creatinine; GFR: Glomerular Filtration Rate. ** *p* < 0.01 * *p* < 0.05.

**Table 3:** AOPPs by renal stage calculated by Cockcroft–Gault, MDRD, CKD-EPI equations.

	Stage 1 GFR ≥90 mL/min/1.73 m^2^	Stage 2 GFR 60–89 mL/min/1.73 m^2^	Stage 3 GFR 30–59 mL/min/1.73 m^2^	Stage 4 GFR 15–29 mL/min/1.73 m^2^	Stage 5 GFR <15 mL/min/1.73 m^2^	*p* *
C–G	133.45 (91.72–186.86)	105.95 (81.35–141.40)	161.75 (120.16–229.38)	532.95 (496.25–566.95)	539.61 (509.56–569.41)	0.0001
MDRD	132.16 (93.47–185.34)	112.13 (84.95–151.41)	176.15 (122.20–229.90)	555.62 (511.53–609.31)	538.99 (505.49–562.69)	0.0001
CKD-EPI	129.30 (93.92–183.99)	113.60 (79.70–158.20)	205.80 (143.20–249.60)	549.95 (505.86–609.61)	538.99 (505.49–562.69)	0.0001

AOPPs: Advanced Oxidative Protein Products; GFR: Glomerular Filtration Rate; C–G: Cockcroft–Gault; MDRD: Modification of Diet in Renal Disease; CKD-EPI: Chronic Kidney Disease Epidemiology Collaboration. * Kruskal Wallis test; *p* < 0.05.

## Data Availability

The participants of this study did not give written consent for their data to be shared publicly, so due to the sensitive nature of the research, supporting data is not available.
